# Explainable Federated Learning for Multi-Class Heart Disease Diagnosis via ECG Fiducial Features

**DOI:** 10.3390/diagnostics15243110

**Published:** 2025-12-07

**Authors:** Tanjila Alam Sathi, Rafsan Jany, AKM Azad, Salem A. Alyami, Naif Alotaibi, Iqram Hussain, Md Azam Hossain

**Affiliations:** 1 Department of Computer Science and Engineering, Islamic University of Technology (IUT), Gazipur 1704, Bangladesh; tanjilaalam187@iut-dhaka.edu (T.A.S.); rafsanjany@iut-dhaka.edu (R.J.); 2 Department of Mathematics and Statistics, Faculty of Science, Imam Mohammad Ibn Saud Islamic University (IMSIU), Riyadh 13318, Saudi Arabia; kazad@imamu.edu.sa (A.A.); saalyami@imamu.edu.sa (S.A.A.); nmaalotaibi@imamu.edu.sa (N.A.); 3 Research Department, King Salman Center for Disability Research, Riyadh 11614, Saudi Arabia; 4 Department of Anesthesiology, Weill Cornell Medicine, Cornell University, New York, NY 10065, USA; iqh4001@med.cornell.edu

**Keywords:** federated learning, explainable AI (XAI), heart disease classification, ECG fiducial features, arrhythmia, ischemia, disability

## Abstract

**Background/Objectives:** Cardiovascular disease (CVD) remains a leading cause of mortality and disability worldwide, with timely diagnosis critical for preventing long-term functional impairment. Electrocardiograms (ECGs) provide essential biomarkers of cardiac function, but their interpretation is often complex, particularly across multi-institutional datasets. **Methods:** This study presents an explainable federated learning framework with long short-term memory (FL-LSTM) for multi-class heart disease classification, capable of distinguishing arrhythmia, ischemia, and healthy states while preserving patient privacy. **Results:** The model was trained and evaluated on three heterogeneous ECG datasets, achieving 92% accuracy, 99% AUC, and 91% F1 score, outperforming existing federated approaches. Model interpretability is provided via SHapley Additive exPlanations (SHAP) and Local Interpretable Model-Agnostic Explanations (LIME), highlighting clinically relevant ECG biomarkers such as P-wave height, R-wave height, QRS complex, RR interval, and QT interval. **Conclusions:** By integrating temporal modeling, federated learning, and interpretable AI, the framework enables secure and collaborative cardiac diagnosis while supporting transparent clinical decision-making in distributed healthcare settings.

## 1. Introduction

Cardiovascular disease (CVD), encompassing a broad range of disorders affecting the heart and blood vessels, remains the foremost contributor to global mortality and disability burden. According to the World Health Organization, CVD accounts for nearly one-third of all deaths worldwide and is also a leading cause of years lived with disability, underscoring its dual impact on survival and long-term health outcomes [1,2]. Beyond mortality, CVD often results in chronic impairment that reduces patients’ functional capacity and quality of life while also creating significant socioeconomic strain on healthcare systems. Among the most prevalent conditions, ischemia, which is caused by inadequate myocardial blood flow, and arrhythmia, which results from irregular electrical activity and is particularly common in the elderly, pose substantial risks of sudden cardiac events and chronic morbidity [3].

Monitoring ECG signals is essential for accurate prognosis and effective management of cardiovascular disease. ECG has become an established diagnostic tool due to its quick, safe, non-invasive, and cost-effective nature [4]. By capturing the heart’s electrical activity, ECG enables the detection of key cardiac abnormalities, including ischemia, arrhythmia, and other cardiovascular disorders. Although modern diagnostic techniques such as echocardiography, cardiac MRI, and coronary angiography provide comprehensive evaluations, they are expensive, time-consuming, and less suitable for continuous or real-time monitoring [5]. The growing incorporation of ECG technology into wearable devices and fitness trackers enables real-time, cost-effective, and accessible monitoring, hence aiding in the early identification, prompt intervention, and long-term treatment of cardiovascular disease [1,6].

Developing accurate and generalizable models for heart disease classification from ECG signals requires large, heterogeneous datasets representing diverse patient populations and clinical settings [7]. However, hospital data often exist in fragmented silos with varying recording systems, protocols, and population-specific biases, leading to covariate shifts that degrade conventional machine learning performance [8]. Additionally, the sensitive nature of ECG data, including its potential use in biometric authentication, limits centralized data sharing. Federated Learning (FL) addresses these challenges by enabling collaborative model training across multiple hospitals while keeping patient data decentralized, improving generalization and mitigating institutional biases compared to single-institution approaches.

Despite these advantages, most FL-based approaches for heart disease classification focus on a single condition, such as arrhythmia or ischemia, limiting their applicability in real-world clinical settings where patients may present with multiple heart conditions [9,10]. Furthermore, many existing FL models employ opaque deep learning architectures that lack interpretability, creating barriers to clinical trust and adoption [11,12,13]. For high-stakes applications such as cardiovascular disease diagnosis, clinicians require not only accurate predictions but also transparent reasoning behind them. Explainable artificial intelligence (XAI) methods address this need by highlighting clinically meaningful ECG features such as the P-wave, QRS complex, QT interval, and ST segment that contribute to diagnostic outcomes [14,15].

Motivated by these challenges, this study presents the first explainable federated learning framework for multi-class heart disease classification. Specifically, we propose a federated long short-term memory (FL-LSTM) model capable of simultaneously classifying arrhythmia, ischemia, and healthy states based on ECG fiducial features. To the best of our knowledge, this is the first effort to combine datasets representing these three heart conditions within a federated and explainable framework. To enhance interpretability, we integrate SHAP [16] and LIME [17], which provide both global and local explanations of model predictions, enabling clinicians to better understand the relevance of specific ECG features. By ensuring decentralized data sharing across multiple hospitals and users, the proposed framework preserves privacy while enabling scalable, real-time monitoring of cardiovascular health. The main contributions of this paper are as follows:

- We propose the first federated learning framework that simultaneously classifies arrhythmia, ischemia, and normal heart function using ECG fiducial markers, addressing a key limitation of prior federated learning approaches that have primarily focused on single disease conditions.
- We integrate explainable XAI techniques (SHAP and LIME) to provide both global and local interpretability of model decisions, facilitating clinical trust and adoption.
- We highlight the practical utility of the proposed framework in enabling privacy-preserving, real-time, and scalable monitoring of cardiovascular health in diverse healthcare environments.

## 2. Literature Review

The electrocardiogram (ECG) is one of the most reliable non-invasive diagnostic tools for assessing cardiac function and detecting abnormalities. ECG analysis typically involves preprocessing to eliminate noise, followed by feature extraction in the temporal or frequency domain to identify fiducial markers such as P-wave, QRS complex, and QT interval. Deviations in these markers—such as ST-segment depression, QT prolongation, or irregular RR intervals—serve as important biomarkers for detecting ischemia and arrhythmia [5,18,19,20]. Accurate interpretation of these fiducial features provides valuable clinical insight into electrical and structural abnormalities of the heart, supporting timely and effective medical intervention.

Recent advances in machine learning (ML) and deep learning (DL) have significantly enhanced the automation of ECG-based disease diagnosis. Classical ML methods, including Support Vector Machines (SVM), K-Nearest Neighbors (KNN), and Artificial Neural Networks (ANN), have demonstrated moderate success using morphological and statistical ECG features extracted from databases such as the European ST-T [21] and MIT-BIH arrhythmia datasets [22,23,24]. Murthy et al. [22] analyzed the European ST-T database and compared ANN, SVM, and KNN for ischemia detection, while Park et al. [23] employed SVM and Kernel Density Estimation (KDE) for the same task. Similarly, the MIT-BIH arrhythmia database [24] has enabled numerous ML-based studies on arrhythmia classification using statistical and morphological ECG features [25,26,27,28]. Ensemble approaches combining Random Forest, KNN, and SVM [29] have also shown improved performance in arrhythmia detection.

Building on these foundations, recent DL architectures such as convolutional neural networks (CNNs), recurrent neural networks (RNNs), and hybrid CNN–LSTM frameworks have further advanced ECG interpretation and heartbeat classification accuracy [30,31,32]. Cardiac arrhythmia, often identified through RR interval irregularities or absent P-waves, serves as a key ECG biomarker for abnormal rhythms [33]. Various deep learning studies have exploited these biomarkers to improve automatic arrhythmia detection, demonstrating significant diagnostic potential. Comprehensive reviews have summarized these advancements and compared the performance of DL models such as CNN, LSTM, and transformer-based networks, while also identifying the gaps that persist in clinical interpretability and generalization [34,35,36,37].

To address the challenges of data centralization and privacy in healthcare analytics, Federated Learning (FL) has emerged as a distributed, privacy-preserving paradigm that enables multiple institutions to collaboratively train models without sharing sensitive patient data, ensuring compliance with data protection standards such as HIPAA [38]. Recent research has explored the feasibility of FL in ECG and cardiac disease classification tasks. Santos et al. [12] implemented a federated CNN architecture on the PhysioNet Challenge dataset [39] for atrial fibrillation detection, achieving an F1 score of 77%. Yaqoob et al. [10] employed a Modified Artificial Bee Colony (MABC) optimization algorithm within an FL framework using the Cleveland Heart Disease dataset [40], reporting 81% accuracy for cardiac prediction. Similarly, Khan et al. [13] utilized an asynchronous deep neural network (DNN) model on UCI datasets [40], achieving 88% accuracy, while Alasmari et al. [11] extended this approach to multimodal cardiac data, reaching 97% accuracy. Zeleke et al. [9] combined Temporal Convolutional Networks (TCN) with attention mechanisms to classify arrhythmias on the MIT-BIH dataset [24], incorporating heatmap-based explanations for interpretability. Furthermore, Agrawal et al. [41] introduced a differential privacy-based FL approach (DP-FedAvg) on the Alberta Health Services dataset, achieving 81% accuracy in distinguishing healthy versus diseased cases.

Despite the growing interest in federated learning for healthcare, current studies remain limited in scope primarily addressing single cardiac conditions such as arrhythmia or ischemia and often lacking explainability mechanisms essential for clinical acceptance. The absence of multi-class classification frameworks and transparent diagnostic interpretation restricts the clinical utility of these models in real-world healthcare environments. Moreover, the integration of biomarker-level interpretability and fiducial feature attribution remains largely unexplored in decentralized ECG analysis. To overcome these limitations, this study introduces a federated long short-term memory (FL-LSTM) model capable of jointly classifying arrhythmia, ischemia, and healthy cardiac states using distributed ECG datasets from multiple institutions. The framework further incorporates XAI methods (SHAP and LIME) to interpret model decisions at both global and local levels, providing clinically meaningful insights into fiducial biomarkers such as P-wave height, R-wave amplitude, QRS duration, and QT interval. This approach bridges the gap between accuracy, privacy preservation, and interpretability, offering a comprehensive, secure, and explainable solution for intelligent cardiovascular diagnosis.

## 3. Materials and Methods

**Figure 1:**
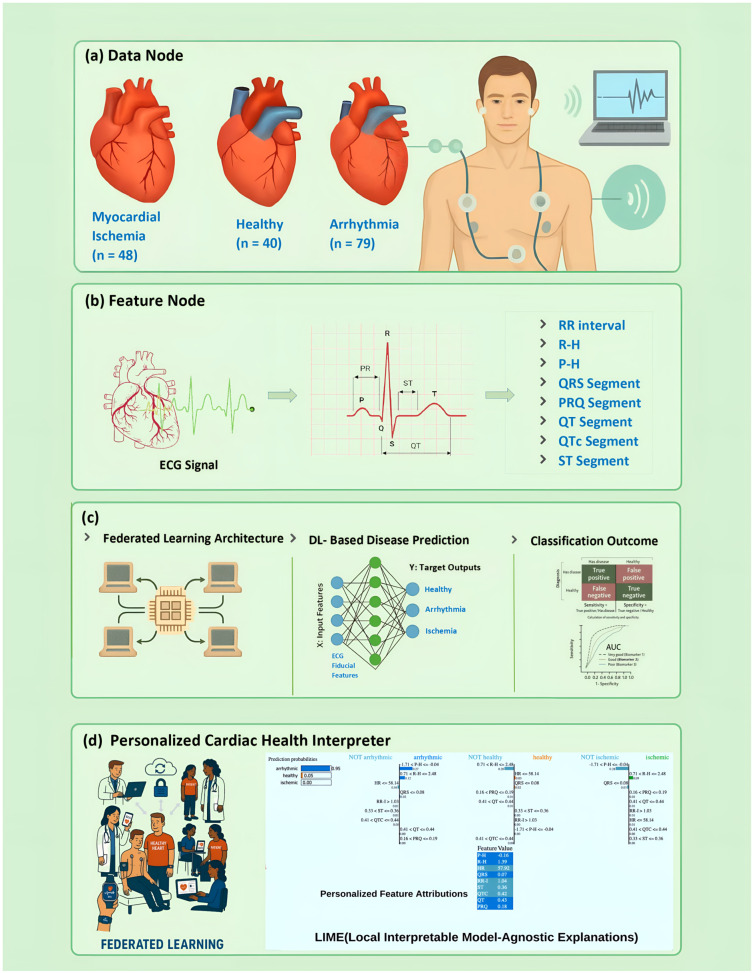
A comprehensive system architecture for arrhythmia, ischemia, and healthy heart condition recognition, employing ECG data alongside interpretable AI techniques. (**a**) Data Acquisition: ECG data collected from arrhythmia, ischemia, and healthy groups. (**b**) Feature Node: Extraction of fiducial features from ECG. (**c**) Model Framework: Utilization of Federated learning with deep learning for prediction of arrhythmia, ischemia, and healthy heart conditions. (**d**) Personalized Diagnostics: Explanation tailored for individual CVD cases.

This section outlines the proposed framework in detail, covering the complete workflow from data acquisition to model interpretation. The process begins with the collection of ECG signals, followed by signal preprocessing and the extraction of clinically relevant fiducial features. The resulting datasets are then integrated for model development. Subsequently, deep learning algorithms are employed for client-side classification, while the Federated Averaging (FedAvg) algorithm is applied at server-side to aggregate local model updates into a global model. The system’s effectiveness is evaluated using standard performance metrics. Finally, explainable artificial intelligence techniques are applied to interpret the model’s predictions, ensuring transparency and clinical trustworthiness. The overall workflow of the proposed study is depicted in Figure 1.

### 3.1. Problem Formulation

The objective of this study is to design a privacy-preserving and interpretable federated learning framework for classifying cardiovascular conditions using ECG data. Consider *N* edge nodes, each corresponding to an institution (hospitals), with datasets denoted as $D_{1},D_{2},\ldots ,D_{N}$. Each dataset consists of ECG signals $E=\{E_{1},E_{2},\ldots ,E_{H}\}$ collected from patients representing arrhythmia, ischemia, and healthy states. Traditional deep learning models, such as LSTM, can be trained on a centralized dataset $D=\{D_{1},D_{2},\ldots ,D_{N}\}$; however, centralization raises major privacy concerns, as ECG data are sensitive and often subject to strict regulatory constraints.

To address this, the proposed framework employs federated learning, where each edge node trains a local LSTM model using its private dataset while keeping raw data decentralized. After several local training epochs, the locally updated parameters $\theta^{i}$ from each client are transmitted to a central server. The server then aggregates these updates using the Federated Averaging (FedAvg) algorithm to update the global model parameters $\theta^{g}$, defined as:

(1) $$\theta^{g}=\frac{1}{N}\sum_{i=1}^{N}\theta^{i}$$

Formally, the federated optimization objective is expressed as:

(2) $$\min_{\theta }\;F\left(\theta \right)=\sum_{i=1}^{N}\frac{|D_{i}|}{\left|D\right|}\;L_{i}\left(\theta \right),$$

where $L_{i}\left(\theta \right)$ represents the local loss of client *i*, $|D_{i}|$ is the size of its dataset, and $\left|D\right|=\sum_{i=1}^{N}\left|D_{i}\right|$ is the total dataset size across all clients. This formulation ensures that clients with larger datasets have proportionally greater influence on the global model.

Finally, to enhance clinical trust, the framework integrates explainable artificial intelligence (XAI) methods. SHAP quantifies the contribution of each fiducial ECG feature (P-wave height, R-wave height, QRS complex, QT interval), while LIME generates instance-level interpretations by approximating the global model locally. These interpretability mechanisms provide both global and local explanations, ensuring transparency and clinical usability.

Overall, the proposed problem formulation enables accurate and interpretable classification of cardiovascular conditions while addressing key challenges such as privacy preservation, institutional heterogeneity, and the need for trustworthy clinical decision support.

### 3.2. ECG Data Acquisition

The study aims to develop a federated learning framework that accurately classifies subjects into arrhythmia, ischemia, and healthy groups. To achieve this, three widely used public ECG databases were utilized:

- MIT-BIH Arrhythmia Database (MIT-BIH) [24]: Includes 47 subjects with 48 half-hour excerpts from 24-h ECG recordings. It is widely recognized as the benchmark dataset for arrhythmia classification tasks. This dataset serves as a representative sample of the diverse ECG waveforms and artifacts that an arrhythmia detector may encounter in routine clinical practice. It encompasses a wide spectrum of arrhythmia as a cardiovascular disease, including complex ventricular, junctional, and supraventricular arrhythmias, as well as conduction abnormalities.
- European ST-T Database [21]: The European ST-T Database (EDB) includes 90 annotated ECG excerpts from 79 subjects (70 men aged 30–84 and 8 women aged 55–71), all diagnosed or suspected with myocardial ischemia, selected to capture a representative range of ECG abnormalities.
- FANTASIA Database [42]: Contains ECG recordings from 40 healthy subjects divided into two groups (young and elderly), providing normal sinus rhythm data for baseline or healthy state analysis.

To provide a clearer comparison, the key characteristics of these datasets are summarized in Table 1. This combined data set provides a balanced representation in three major categories, arrhythmia, ischemia, and healthy, allowing for comprehensive training and evaluation of the proposed federated learning framework.

### 3.3. Data Preprocessing

To ensure consistency across datasets with different acquisition settings, all ECG recordings were first resampled to a uniform frequency of 200 Hz. This rate is recommended by standard QRS-detection algorithms and ensures comparable temporal precision for the calculation of fiducial intervals such as RR, PR, and QT [15].

Each recording was segmented beat-by-beat using automated detection, and noisy or corrupted portions were removed prior to feature extraction. “Premature, missing, or ectopic” beats refer to those flagged as abnormal by the detection algorithm; these samples were excluded so that only valid beats contribute to the final fiducial dataset. After filtering, the cleaned signals were passed to the feature-extraction module described next.

### 3.4. Fiducial Feature Extraction

ECG feature extraction in this study consists of both fiducial features and heart rate variability (HRV) features, as illustrated in Figure 1b. Fiducial features were obtained through the identification of onset, offset, and peak points of the standard P–QRS–T wave profile, while HRV features were derived from ECG signals analyzed in the time domain. To ensure consistency, only one channel was selected from each recording, and all signals were resampled at 200 Hz to match the optimized rate for QRS detection algorithms. Premature, missing, or ectopic beats were filtered out to minimize noise and improve reliability. Fiducial features were extracted on a cycle-by-cycle basis, capturing both time and voltage measurements of the P-wave, Q-wave, and S-wave events, as well as the duration of QRS events across different intervals of the ECG waveform. AcqKnowledge version 5.0 (Biopac Systems Inc., Goleta, CA, USA) was used for this purpose, offering interactive real-time analysis and visualization. AcqKnowledge 5.0 automatically preprocesses ECG signals, detects fiducial points (P-wave, QRS complex, T-wave), and extracts clinically relevant features such as RR intervals, wave amplitudes, PR, QT/QTc intervals, QRS duration, and ST-segment deviations for comprehensive cardiac analysis.

A number of clinically significant reference features were derived from the ECG recordings, including heart rate based on the RR interval, amplitudes of the P- and R-waves, and temporal intervals such as PR, QT, and corrected QT (QTc). Additional markers such as QRS duration and ST-segment were also extracted to capture cardiac conduction and repolarization characteristics. Together, these fiducial and HRV features provide a comprehensive representation of the cardiac cycle, supporting reliable disease classification. Detailed definitions of these features can be found in [15].

The total number of fiducial features obtained for each category is as follows: 581,219 for ischemic cases, 267,360 for healthy cases, and 40,238 for arrhythmic cases. Each record includes nine fiducial characteristics derived from the ECG waveform, namely heart rate (HR), R-wave height (R-H), P-wave height (P-H), PR interval, QT interval, corrected QT interval (QTc), RR interval, QRS duration, and ST segment. These features collectively capture both the temporal and amplitude-based characteristics of the P–QRS–T cycle, providing clinically meaningful parameters for robust disease classification.

### 3.5. Dataset Integration and Class Imbalance Handling

A uniform method was used to extract features from three separate databases, and then the datasets were combined and merged. The combined dataset exhibited substantial class imbalance; therefore, to mitigate this issue and improve model robustness, the Synthetic Minority Over-Sampling Technique (SMOTE) [43] was applied during training. Following data augmentation, the merged dataset comprised a total of 1,102,491 ECG fiducial samples, including 111,659 samples from individuals with arrhythmic disorders, 705,983 samples from patients with ischemic heart disease, and 284,849 samples from healthy subjects.

### 3.6. Federated Learning Architecture

Federated Learning (FL) is a decentralized paradigm that enables collaborative model training across distributed clients without sharing raw data. Each client trains a local model using its private dataset, and only the learned parameters are communicated to a central server. The server then aggregates these updates to form a global model, which is redistributed to clients for further training. In this work, we adopt the standard FedAvg algorithm as the aggregation mechanism due to its proven stability, computational efficiency, and strong empirical performance across heterogeneous medical datasets, making it well suited for ECG-based federated environments where client data distributions are naturally non IID. This section presents the working principles of the proposed FL architecture through the client-side pseudocode, which describes local training, and the server-side pseudocode, which outlines the aggregation and update of the global model.

#### 3.6.1. Local Model Training at Client Nodes

The client-side pseudocode outlines the local training process executed independently on each client device. In federated learning, clients collaboratively train a shared model by performing computations locally on their private datasets without transferring raw data to the central server, thereby ensuring data privacy and security. Algorithm 1 presents the client-side training procedure adopted in the proposed framework, detailing how each client iteratively updates its local model parameters before contributing to the global aggregation process.

- Input Parameters: Each client *i* begins with its own dataset $D^{i}$, a pre-defined batch size *B*, local epochs *E*, learning rate η, and model parameters $\theta^{i}$, which are randomly initialized at the start.
- Epoch Loop: The training process on each client proceeds for *E* local epochs. In each epoch, the client divides its dataset $D^{i}$ into mini-batches of size *B*, which are used in the training process.
- Mini-Batch Training: For each mini-batch *b*, the client computes a forward pass, denoted as $h^{i}=f\left(x^{i},\theta^{i}\right)$, where $x^{i}$ represents the input data and *f* is the model function parameterized by $\theta^{i}$. Then, the loss $L(y^{i},{\hat{y}}^{i})$ is computed between the predicted values ${\hat{y}}^{i}$ and the actual ground truth $y^{i}$, from which the gradients of the loss with respect to the model parameters are calculated as $\Delta \theta^{i}=\frac{\partial L(y^{i},{\hat{y}}^{i})}{\partial \theta^{i}}$.
- Model Update: After computing the gradients, the client updates its local model parameters using stochastic gradient descent (SGD). The updated model parameters are obtained using the rule $\theta^{i}\leftarrow \theta^{i}-\eta \cdot \Delta \theta^{i}$, where η is the learning rate that controls how much the model parameters should change with each gradient step.
- Sending Updated Parameters: Once all local epochs are completed, the client sends the updated model parameters $\theta^{i}$ to the central server for aggregation.

**Algorithm 1** Client-Side Pseudocode
**Require:** Client dataset $D^{i}$, Batch size *B*, Local epochs *E*, Learning rate η, Model parameters $\theta^{i}$**Ensure:** Updated local model $\theta^{i}$1:Initialize: Random weights $\theta^{i}=\mathrm{rand}\left(\right)$2:**for** epoch e=1 to *E* **do**3:       Divide $D^{i}$ into mini-batches of size *B*4:       **for** each mini-batch *b* **do**5:             Compute forward pass $h^{i}=f\left(x^{i},\theta^{i}\right)$6:             Compute gradients $\Delta \theta^{i}=\frac{\partial L(y^{i},{\hat{y}}^{i})}{\partial \theta^{i}}$7:             Update local model $\theta^{i}\leftarrow \theta^{i}-\eta \cdot \Delta \theta^{i}$8:       **end for**9:
**end for**
10:Send updated model parameters $\theta^{i}$ to the server


#### 3.6.2. Global Model Aggregation at the Central Server

The server-side pseudocode defines the central coordination mechanism in the federated learning process. The server is responsible for aggregating local model updates received from multiple clients and updating the global model accordingly. This ensures consistent model improvement while preserving data locality at the client side. After each aggregation, the updated global model is redistributed to all participating clients for further local training. Once the final global model is obtained, model interpretability techniques such as SHAP and LIME are applied to enhance transparency and trustworthiness. SHAP quantifies the contribution of each feature to the model’s predictions, while LIME provides instance-specific explanations by locally approximating the model’s decision boundary. These techniques collectively enable the proposed framework to generate interpretable and explainable diagnostic insights from ECG data. Algorithm 2 presents the complete workflow of the server-side operations employed in the proposed federated learning framework.
**Algorithm 2** Global Server-Side Pseudocode**Require:** Model parameters from clients $\theta^{1},\theta^{2},\ldots ,\theta^{N}$, Aggregation function FedAvg**Ensure:** Global model parameters $\theta^{g}$1:Initialize: Global model $\theta^{g}=\mathrm{rand}\left(\right)$2:**for** each round r=1 to *R* **do**3:      **for** each client *i* **do**4:            Receive local model $\theta^{i}$5:      **end for**6:      Aggregate local models $\theta^{g}=\frac{1}{N}\sum_{i=1}^{N}\theta^{i}$7:**end for**8:Deploy the global model $\theta^{g}$9:**Apply SHAP and LIME** on the final global model $\theta^{g}$10:Compute SHAP values $\phi_{j}\left(x\right)=\frac{\partial f(x)}{\partial x_{j}}$ for feature attribution11:Use LIME for instance-wise explanations E(x)≈f(x)12:Analyze and interpret model predictions using SHAP and LIME explanations

- Input Parameters: The server receives local model parameters $\theta^{1},\theta^{2},\ldots ,\theta^{N}$ from *N* clients and uses an aggregation function, such as Federated Averaging (FedAvg), to combine the updates.
- Global Model Initialization: At the start, the server initializes a global model $\theta^{g}$ with random weights.
- Aggregation Loop: For each round r=1 to *R*, the server collects the local model parameters $\theta^{i}$ from each client. It then aggregates these parameters to update the global model using the FedAvg algorithm. The global model parameters $\theta^{g}$ are updated as the average of the client models:
  $$\theta^{g}=\frac{1}{N}\sum_{i=1}^{N}\theta^{i}$$
  where *N* is the number of participating clients.
- Global Model Deployment: After each aggregation, the server deploys the updated global model back to the clients for the next round of local training.
- Model Interpretability: Once the final global model is obtained, model interpretability techniques such as SHAP and LIME are applied. SHAP values are computed to quantify the contribution of each feature $x_{j}$ to the model’s prediction, represented as:
  $$\phi_{j}\left(x\right)=\frac{\partial f(x)}{\partial x_{j}}$$
  These values help explain the importance of individual features in the model’s decision-making process. LIME is also used for instance-wise explanations by approximating the global model f(x) locally for specific data points, providing interpretable insights into how the model behaves on specific instances. This allows us to analyze and interpret model predictions with a focus on transparency and explainability.

#### 3.6.3. Client-Side Deep Learning Architecture Using LSTM

In this study, a Long Short-Term Memory (LSTM) network was selected as the core deep learning model for client-side training due to its proven ability to capture temporal dependencies in physiological signals, as shown in Figure 1c. The LSTM architecture, one of the top-performing models in the PhysioNet 2020 Challenge [42], is particularly effective for modeling the dynamic nature of cardiac rhythm variations over time, enabling robust detection and classification of arrhythmia, ischemia, and normal heart conditions [15,43]. Unlike conventional feedforward neural networks, LSTM incorporate feedback connections that allow information to persist across time steps, thereby mitigating issues of vanishing or exploding gradients commonly encountered in traditional recurrent neural networks (RNNs). Each memory cell in the LSTM comprises an input gate, output gate, and forget gate, which collectively regulate the internal flow of information and maintain long-term contextual memory relevant to cardiac signal sequences.

**Figure 2:**
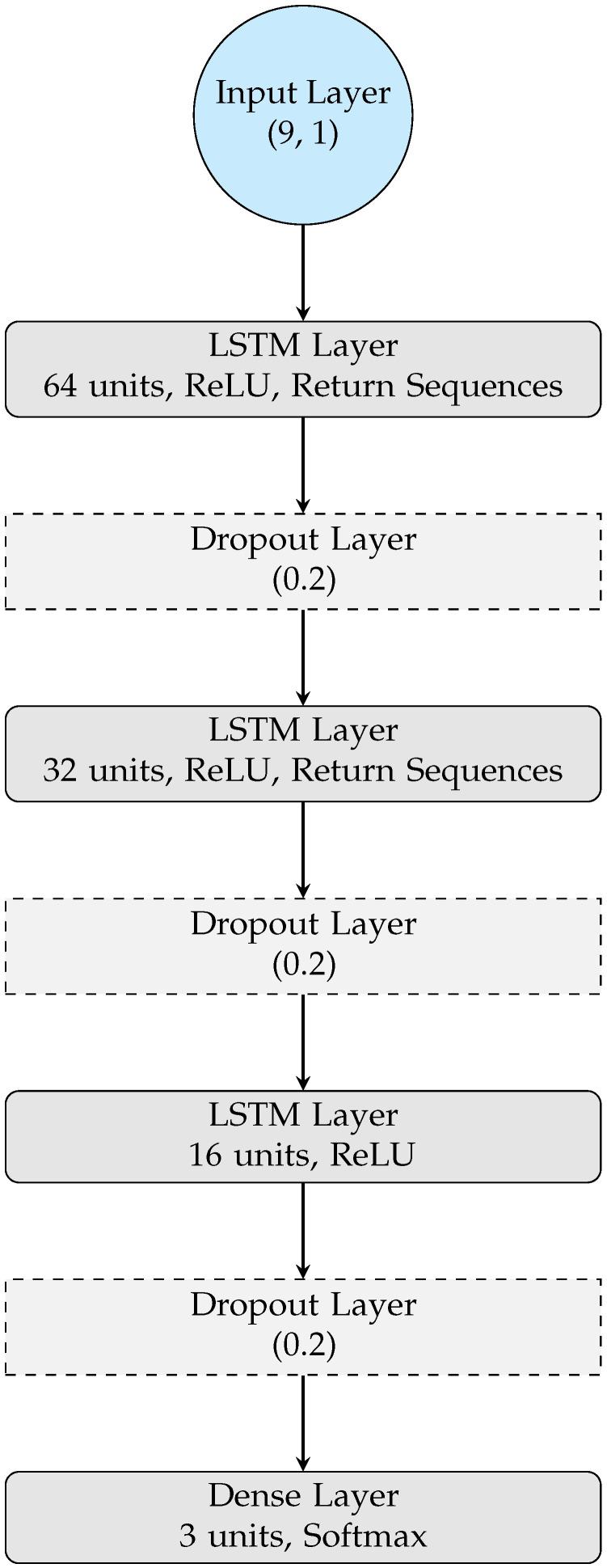
LSTM Model Architecture.

The proposed architecture of the LSTM model comprises multiple stacked LSTM layers, interleaved with dropout layers to prevent overfitting, and a dense output layer for final classification, as illustrated in Figure 2. The input layer is designed with neurons corresponding to the extracted ECG fiducial features, whereas the output layer consists of neurons equal to the number of target classification categories. Through extensive fine-tuning, the Rectified Linear Unit (ReLU) and SoftMax activation functions were found to yield optimal performance in feature representation and class probability estimation. This configuration allows the model to effectively learn both short-term and long-term dependencies in ECG sequences, enabling accurate and reliable classification across distributed healthcare clients without compromising data privacy. The detailed structure and layer-wise configuration of the proposed LSTM model are outlined below:

- Input Layer: The input layer takes data in the shape of (9, 1), representing 9 time steps with 1 feature per time step. This input format is typical for sequential data such as sensor readings or biomedical time-series data.
- LSTM Layer 1: The first LSTM layer has 64 units with a ReLU activation function and is configured to return sequences. Returning sequences is important because it allows the output of this LSTM layer to be passed to the next LSTM layer while preserving the sequence structure. This layer is crucial for learning temporal dependencies in the data, as LSTM units are specifically designed to capture long-term dependencies in time-series data.
- Dropout Layer 1: After the first LSTM layer, a dropout layer with a rate of 0.2 is applied to prevent overfitting. In federated learning, each client’s local data might be limited or highly variable, so dropout ensures the model generalizes well across different clients.
- LSTM Layer 2: The second LSTM layer has 32 units, also using the ReLU activation function and returning sequences. Reducing the number of units in this layer helps in gradually compressing the learned representations while still maintaining the temporal relationships.
- Dropout Layer 2: A second dropout layer with the same rate (0.2) follows the second LSTM layer, further ensuring that the model does not overfit, especially on smaller client datasets.
- LSTM Layer 3: The third LSTM layer has 16 units with a ReLU activation function, but it does not return sequences. Instead, it outputs a single vector that summarizes the learned features from the previous LSTM layers. This layer acts as a final compression of the temporal features before they are passed to the output layer.
- Dropout Layer 3: Another dropout layer with a rate of 0.2 is applied after the third LSTM layer to maintain regularization.
- Output Layer: The final layer is a dense layer with 3 units and a softmax activation function. This indicates that the model is designed for multi-class classification, where the output is a probability distribution over 3 classes arrhythmic, healthy, ischemic in a medical context). The class with the highest probability will be chosen as the predicted output.

#### 3.6.4. Explainable Artificial Intelligence (XAI) Techniques for Model Interpretability

To ensure the interpretability and clinical reliability of the proposed Federated Learning framework, two complementary model-agnostic explanation techniques were adopted: SHAP [16] and LIME [17]. Both approaches are widely used in biomedical machine learning because they can explain predictions from any model architecture without requiring modifications to its internal structure.

SHAP quantifies each feature’s contribution to the model output through an additive decomposition derived from cooperative game theory. For a given instance, the Shapley value for feature *i* is computed as:

(3) $$\phi_{i}=\sum_{S\subseteq F\backslash \{i\}}\frac{|S|!(|F|-|S|-1)!}{|F|!}\;\left(f_{S\cup \{i\}}\left(x_{S\cup \{i\}}\right)-f_{S}\left(x_{S}\right)\right),$$

where *F* denotes the complete set of input features, *S* is a subset of *F* excluding feature *i*, $f_{S}\left(x_{S}\right)$ represents the model output when only the features in *S* are available, and $f_{S\cup \{i\}}\left(x_{S\cup \{i\}}\right)-f_{S}\left(x_{S}\right)$ measures the marginal contribution of feature *i*. The factorial coefficient assigns a weight that averages this contribution across all possible subsets, producing a fair global attribution $\phi_{i}$ for each fiducial feature.

LIME constructs a simple surrogate model around each individual prediction to locally approximate the decision boundary of the complex model. For an instance *x*, LIME finds an interpretable model g∈G that minimizes the following objective:

(4) $$\xi \left(x\right)=\arg\min_{g\in G}\left[L(f,g,x)+\Omega (g)\right],$$

where *f* is the original FL-LSTM model, and *g* is a candidate interpretable model (a sparse linear model). The loss term L(f,g,x) measures the local fidelity of *g* to *f* in the neighborhood of *x*, while Ω(g) penalizes model complexity to preserve interpretability. In practice, $L\left(f,g,x\right)=\sum_{z,z^{\prime }\in Z}\pi_{x}\left(z\right)\;{\left(f\left(z\right)-g\left(z^{\prime }\right)\right)}^{2}$, where *Z* is a set of perturbed samples generated around *x*, and $\pi_{x}\left(z\right)$ is a kernel function assigning higher weights to samples closer to *x* in feature space.

We focus on SHAP [16] and LIME [17] because both are model-agnostic and operate effectively on tabular inputs such as ECG fiducial features. Other gradient-based methods such as Grad-CAM, Integrated Gradients, or DeepLIFT are primarily designed for convolutional networks that process raw images or waveforms, and are therefore less suited to the structured, low-dimensional feature space used in this study. SHAP provides a global understanding of which fiducial variables most influence the FL-LSTM model’s behavior, whereas LIME delivers local explanations for individual predictions, together forming a clinically transparent interpretability layer within the federated learning pipeline.

### 3.7. Experimental Setup

The proposed heart disease classification framework was implemented and evaluated in a controlled experimental environment.

#### 3.7.1. Computational Environment

All experiments were performed on Google Colab using the PyTorch 2.3.0 deep learning framework. The hardware configuration consisted of an NVIDIA Tesla T4 GPU with 15 GB GPU memory and CUDA 12.2, supported by an Intel Xeon CPU (2 cores, 2.30 GHz) and 13 GB of system RAM. This setup is representative of a mid-range server or workstation and is sufficient for real-time inference. Table 2 presents the detailed experimental setup.

#### 3.7.2. Train–Test Split and Federated Clients

After preprocessing and feature extraction, the merged fiducial dataset was first split at the subject level into 67% training and 33% testing. In each communication round, 10 clients participated in the federated learning process, with each client receiving a maximum of 10% of the training data (approximately 6.7% of the entire dataset). The held-out test set was never used for model selection or hyperparameter tuning and serves as an independent set for evaluating generalization performance. The training portion was further partitioned into 10 non-overlapping shards, each assigned to a virtual client representing an institution (hospitals). To emulate realistic institutional heterogeneity, these shards were non-IID in terms of class proportions (arrhythmia, ischemia, healthy) and sample size, so that some clients were arrhythmia-dominant, others ischemia-dominant, etc. In every communication round, all 10 clients participated in training.

#### 3.7.3. Federated Training Procedure

Federated learning was implemented using a custom PyTorch 2.3.0 training loop without relying on a dedicated FL framework such as TensorFlow Federated or PySyft. Each communication round consisted of:

1. The central server broadcasting the current global LSTM model parameters to all clients.
2. Each client performing local training for a fixed number of epochs on its private dataset.
3. Clients sending their updated model parameters back to the server.
4. The server aggregating the received parameters using the Federated Averaging (FedAvg) algorithm to obtain new global parameters.

#### 3.7.4. Hyperparameters and Cross-Validation

On each client, we used a mini-batch size of 32, 15 local epochs, a learning rate of 0.001, the Adam optimizer, and sparse categorical cross-entropy as the loss function. To assess robustness, we performed a federated 10-fold cross-validation within the training set: the training portion was divided into ten folds, nine folds were used for federated training, and the remaining fold was used as a validation fold in each iteration (Algorithm 3). After selecting the best-performing configuration based on validation metrics, the final FL-LSTM model was retrained on the full training set and evaluated once on the held-out test set.
**Algorithm 3** Federated 10-Fold Cross-Validation Procedure1:Partition global dataset *D* into folds $\left\{F_{1},F_{2},\ldots ,F_{10}\right\}$2:**for** k=1 to 10 **do**3:      Assign $F_{k}$ as the test fold; remaining folds as training data4:      **for** each client $c_{i}$ in participating clients **do**5:            Train local model $M_{i}^{\left(k\right)}$ on local training subset6:            Send local parameters $\theta_{i}^{\left(k\right)}$ to the central server7:      **end for**8:      Aggregate global model: $\theta^{\left(k\right)}=\mathrm{Aggregate}\left(\theta_{1}^{\left(k\right)},\ldots ,\theta_{n}^{\left(k\right)}\right)$9:      Evaluate global model on test fold $F_{k}$10:**end for**11:Compute mean performance metrics across all folds

#### 3.7.5. Batch Size, Epochs, and Learning Rate

A batch size of 32 was adopted following the mini-batch gradient descent approach. The model was trained over 15 epochs, with each epoch comprising 30 client updates. The learning rate (LR) was initialized at 0.001 to ensure stable convergence.

#### 3.7.6. Optimizer and Loss Function

The Adam optimizer [44] was utilized to accelerate convergence and efficiently manage noisy gradient estimations. Adam’s adaptive learning capabilities make it well-suited for large-scale and distributed training scenarios such as federated learning. The sparse categorical cross-entropy loss function was employed, which extends the conventional categorical cross-entropy to handle multiple label classes, ensuring effective optimization in the multi-class classification task.

## 4. Result Analysis

This section presents a comprehensive evaluation of the experimental results obtained from the heart disease classification task using the proposed Federated Learning (FL) framework. We first report the statistical analyses used to evaluate the model significance and stability across multiple runs. Following this, model performance is examined using standard evaluation metrics, including accuracy, precision, recall, F1-score, and AUC-ROC, alongside an analysis of the impact of communication rounds on global model convergence. Finally, model interpretability is explored through XAI techniques—specifically SHAP and LIME to provide clinically meaningful insights into feature contributions and the reasoning behind model predictions.

### 4.1. Performance Evaluation Metrics

The performance of the heart disease classification models is evaluated using standard metrics derived from the confusion matrix. The confusion matrix consists of the counts of True Positives (TP), True Negatives (TN), False Positives (FP), and False Negatives (FN). Using these values, we calculate the following metrics:

- Accuracy: Measures the proportion of correctly classified instances among all instances.
  (5) $$\mathrm{Accuracy}=\frac{TP+TN}{TP+TN+FP+FN}$$
- Precision: Indicates the proportion of correctly predicted positive instances among all predicted positives.
  (6) $$\mathrm{Precision}=\frac{TP}{TP+FP}$$
- Recall (Sensitivity): Measures the proportion of correctly predicted positive instances among all actual positives.
  (7) $$\mathrm{Recall}=\frac{TP}{TP+FN}$$
- F1 Score: The harmonic mean of precision and recall, providing a balance between the two.
  (8) $$F1=2\cdot \frac{\mathrm{Precision}\cdot \mathrm{Recall}}{\mathrm{Precision}+\mathrm{Recall}}$$
- Area Under the Curve (AUC): Represents the area under the Receiver Operating Characteristic (ROC) curve, reflecting the trade-off between True Positive Rate and False Positive Rate.
- False Negative Rate (FNR): The proportion of positive instances incorrectly classified as negative.
  (9) $$\mathrm{FNR}=\frac{FN}{TP+FN}$$
- False Positive Rate (FPR): The proportion of negative instances incorrectly classified as positive.
  (10) $$\mathrm{FPR}=\frac{FP}{FP+TN}$$
- Loss: Measures the discrepancy between the predicted and actual labels, here calculated using sparse categorical cross-entropy.
- Misclassification Rate (MC Rate): Represents the proportion of incorrect predictions among all predictions.
  (11) $$\mathrm{MC}\;\mathrm{Rate}=\frac{FP+FN}{TP+TN+FP+FN}$$

These metrics together provide a comprehensive assessment of model performance, considering both overall accuracy and class-wise prediction reliability.

### 4.2. Statistical Analysis

To evaluate the robustness and statistical reliability of the proposed framework, all experiments were repeated under a federated 10-fold cross-validation scheme [45,46]. In each fold, nine partitions were used for federated training and one for validation, following the setup described in Section 3.7. After model selection, the final FL-LSTM configuration was retrained on the entire training set and evaluated once on the held-out test set to estimate unbiased generalization performance [47].

For every fold, the following metrics were computed on both validation and test data: Accuracy, Precision, Recall (Sensitivity), Specificity, F1-score, and Area Under the ROC Curve (AUC) [48,49]. Mean and standard deviation values across folds are reported in Table 3 to quantify variability due to data partitioning [45]. Where appropriate, statistical significance between the FL-LSTM model and baseline centralized or non-federated LSTM architectures was examined using a paired *t*-test (p<0.05) [50]. This analysis confirms that improvements in Accuracy, F1-score, and AUC achieved by the federated model are statistically significant and not due to random variation [51].

### 4.3. Performance Evaluation and Analysis of Proposed FL-LSTM Model

A comprehensive evaluation of the proposed Federated Learning-based Long Short-Term Memory (FL-LSTM) model for heart disease classification was conducted. The model’s performance was assessed using multiple evaluation metrics, including accuracy, F1 score, false negative rate (FNR), false positive rate (FPR), loss, misclassification rate (MC rate), precision, and recall, over 15 communication rounds. The detailed results are summarized in Table 4. Overall, the FL-LSTM model shows steady performance gains throughout training, demonstrating its ability to effectively capture temporal dependencies in ECG sequences while preserving data privacy in a distributed environment.

The model’s accuracy increases consistently over the training rounds, reaching 90.91% in Round 15, which reflects strong generalization capability on heterogeneous client data. The F1 score attains 90.88%, confirming balanced precision and recall despite client-level data variability. Similarly, the FNR decreases steadily to 0.0347, demonstrating that the model effectively minimizes false negatives, which is particularly critical in clinical diagnosis. The FPR also reduces to 0.0757, indicating an improved ability to distinguish between healthy and abnormal ECG patterns.

A consistent decline in loss is observed throughout training, with the final loss value recorded at 0.2446, signifying improved convergence and model stability. The misclassification rate (MC rate) shows a continuous decrease over successive rounds, reaching 0.0909 in Round 15. This result aligns with the accuracy improvements and further reinforces the model’s effectiveness in making correct predictions. Additionally, both precision and recall exhibit strong performance, reaching 0.9101 and 0.9091, respectively, by the final round. These values collectively demonstrate that the FL-LSTM model maintains a high balance between sensitivity and specificity across clients.

In summary, the FL-LSTM model achieves superior performance across all key evaluation metrics, including a notably low misclassification rate, confirming its reliability for accurate and privacy-preserving heart disease classification in distributed healthcare environments.

#### 4.3.1. Convergence Behavior Under Varying Communication Rounds

**Figure 3:**
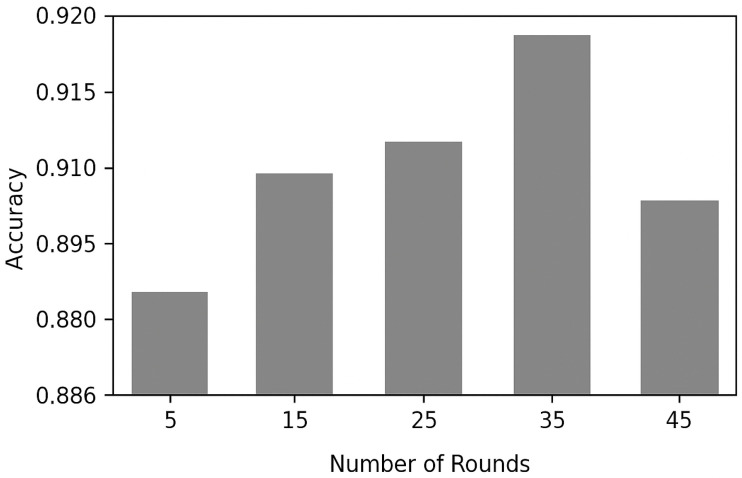
FL-LSTM Number of Communication Round VS Accuracy.

We examine the effect of communication rounds on the precision of FL-LSTM models. As shown in Figure 3, performance improves with increased communication due to more effective aggregation of local models. Notably, after 35 rounds, the model achieves 92% accuracy, with gains plateauing beyond 35–40 rounds, indicating rapid convergence and diminishing returns from additional communication.

#### 4.3.2. Discriminative Performance Evaluation via AUC-ROC

**Figure 4:**
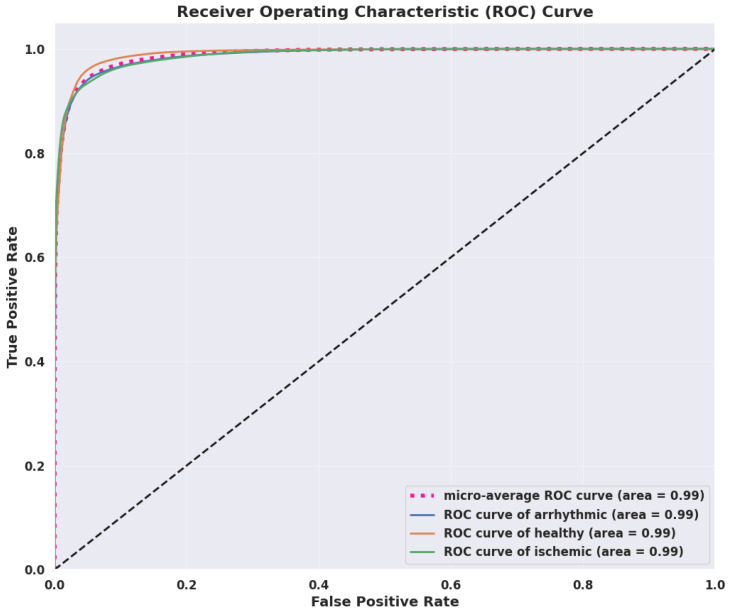
Receiver Operating Characteristic (ROC) curves with AUC scores for the three classes: arrhythmic, healthy, and ischemic of the FL-LSTM model.

The ROC curve evaluates the trade-off between true positive and false positive rates across classification thresholds, with the AUC reflecting overall discriminative performance. An AUC of 1 indicates perfect class separation, while 0 denotes complete misclassification. As illustrated in Figure 4, the FL-LSTM model achieves near-perfect AUC scores of 0.99 for arrhythmic, healthy, and ischemic classes, highlighting its exceptional ability to distinguish between positive and negative instances.

#### 4.3.3. Explaining FL-LSTM Decisions Through Fiducial Feature Influence via SHAP

**Figure 5:**
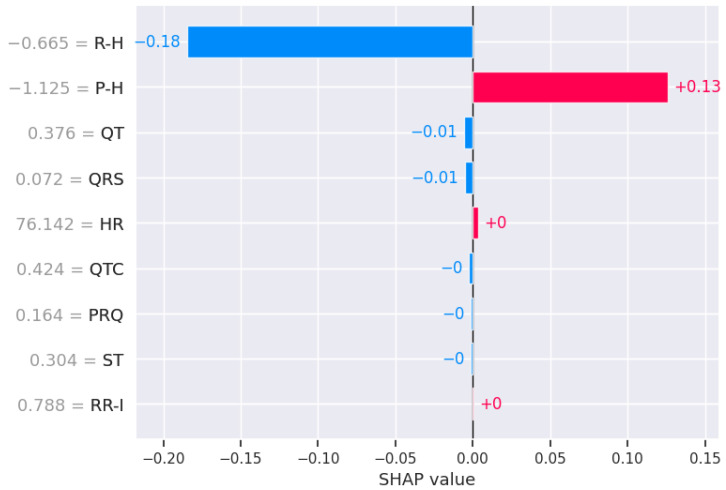
SHAP summary plot for the FL-LSTM model illustrating the influence of fiducial ECG features on model predictions. Features such as R–H and P–H show the strongest negative and positive impacts, respectively, while others contribute minimally, as indicated by SHAP values near zero.

To interpret the decision-making process of the FL-LSTM model, SHAP analysis was applied to the testing dataset to quantify the relative contribution of fiducial ECG features to the model’s predictions. Figure 5 presents the SHAP summary plot, illustrating the mean absolute SHAP values for each feature. The SHAP values along the horizontal axis indicate both the magnitude and direction of each feature’s impact, where positive values increase and negative values decrease the predicted output. Among the fiducial features, R–H exhibits the most significant negative influence with a SHAP value of −0.18, whereas P–H provides the strongest positive contribution with a SHAP value of +0.13 Other features, including QT, QRS, HR, QTC, PRQ, ST, and RR–I, show minimal contributions with SHAP values near zero, suggesting limited influence on the model’s predictive outcomes. This analysis highlights the dominance of specific fiducial intervals in guiding the model’s classification decisions.

#### 4.3.4. Explaining FL-LSTM Decisions Through LIME Visualization

**Figure 6:**
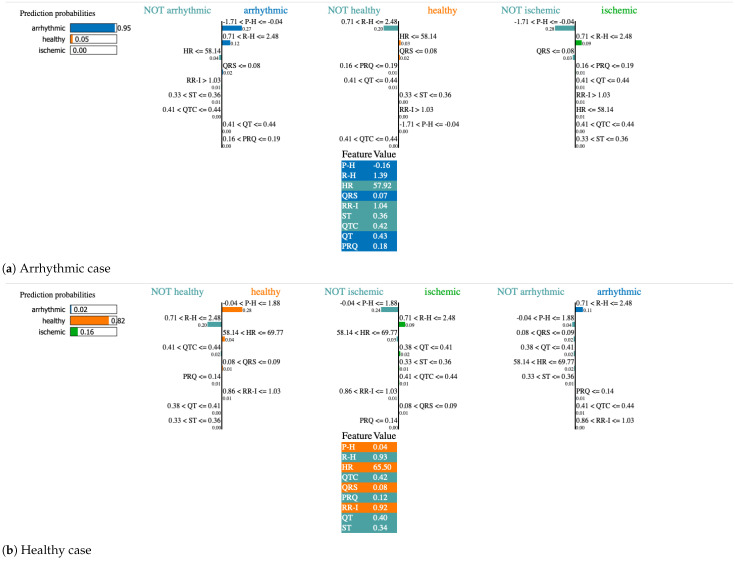

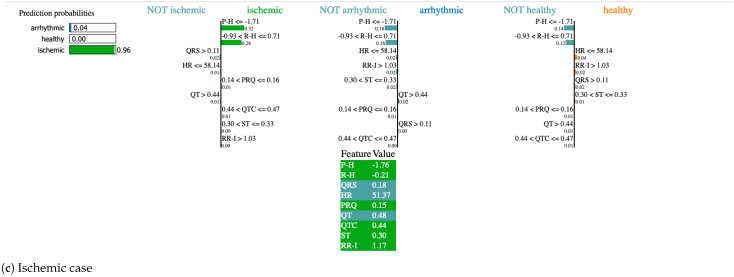
Individualized interpretation of ECG-based heart disease predictions using the LIME [17] approach with the FL-LSTM model. The three representative cases-(**a**) arrhythmic, (**b**) healthy, and (**c**) ischemic-highlight the most influential fiducial features (colored cells) and their associated prediction probabilities. Bar length denotes feature contribution, while the colors (blue, orange, and green) correspond to arrhythmic, healthy, and ischemic predictions, respectively.

To further enhance the interpretability of the FL-LSTM model, the Local Interpretable Model-agnostic Explanations (LIME) technique was employed to provide individualized explanations of ECG-based heart disease predictions. Figure 6a–c presents LIME visualizations for three representative cases—arrhythmic, healthy, and ischemic—selected from the testing dataset (row-3, row-45, and row-95, respectively). The LIME framework generates local surrogate models to approximate the complex FL-LSTM decision boundaries, thereby revealing the relative contribution of key fiducial ECG features to each specific prediction. The visualization uses horizontal bars to indicate class probabilities, ranging from 0 to 1, with longer bars representing higher likelihoods. The bar colors correspond to the predicted class: blue for arrhythmic, orange for healthy, and green for ischemic conditions.

In the arrhythmic case, the model predicted probabilities of 0.95 for arrhythmic, 0.05 for healthy, and 0.00 for ischemic, with influential features including P-wave height (P–H), R-height (R–H), QRS complex, and QT interval as shown in Figure 6a. When it comes to arrhythmia prediction, LIME further emphasized the importance of fiducial features such as P–H, R–H, QRS complex, and QT interval across all evaluated models. For instance, P–H (27%), R–H (12%), QRS, and QT were identified as the dominant contributors, collectively resulting in the high predicted probability of 0.95 for the arrhythmic class.

For ischemia prediction, the models consistently highlighted P-wave height (P–H), R-wave height (R–H), QTc interval, and RR interval (RR–I) as the most influential determinants of classification. Specifically, P–H (32%), R–H (28%), QTc (10%), and RR–I emerged as the top contributing features, collectively driving a 96% prediction probability for ischemia as illustrated in Figure 6c. These findings suggest that the models effectively capture the morphological and temporal ECG variations associated with ischemic conditions, reinforcing the physiological relevance and interpretability of the learned feature representations.

For the healthy case, the model yielded a prediction probability of 82% for healthy, 16% for ischemic, and 2% for arrhythmic conditions. The most influential fiducial features contributing to this prediction were P-wave height (P–H), heart rate (HR), RR interval (RR–I), and the QRS complex, as illustrated in Figure 6b. These features collectively capture the electrophysiological stability and regularity characteristic of healthy cardiac function, demonstrating the model’s ability to accurately distinguish normal ECG patterns from pathological variations.

## 5. Discussion

This study is the first to employ a federated learning framework capable of classifying three major cardiac conditions: arrhythmia, ischemia, and healthy states. The comparative results presented in Table 5 indicate that the proposed FL-LSTM model performs more effectively than other existing federated learning approaches for ECG based heart disease classification. Unlike previous studies such as those by Yaqoob et al. [10], Khan et al. [13], and Agrawal et al. [41], which mainly focused on binary classification or generalized cardiac prediction, the proposed model addresses multi class prediction with clinical significance. The model was developed and evaluated using three heterogeneous datasets collected from diverse clinical and experimental sources, ensuring robustness and cross domain generalization. The proposed FL LSTM achieved an accuracy of 92%, an AUC of 99%, and an F1 score of 91%, surpassing comparable methods such as DP-FedAvg [41] and TCN Attention models [9]. These findings highlight the capability of the model to capture complex temporal relationships in ECG signals while preserving data privacy through the Federated Averaging (FedAvg) approach.

Beyond classification performance, the interpretability of our model represents a major advancement toward trustworthy AI in clinical cardiology. The integration of explainable AI techniques provides transparency into the decision-making process, bridging the gap between computational accuracy and clinical relevance. The analysis revealed that fiducial ECG features such as P-wave height (P–H), R-wave height (R–H), QTc interval, and RR interval (RR–I) significantly influenced classification outcomes. For arrhythmia prediction, SHAP values highlighted P–H (27%) and R–H (12%) as the most influential predictors, whereas ischemia prediction was strongly driven by P–H (32%) and R–H (28%). For healthy cases, the model associated high probabilities with balanced patterns in HR, RR–I, and QRS complex morphology. These findings align with established clinical literature [15] linking variations in P–H and QTc to atrial and ventricular conduction abnormalities, supporting the physiological validity of the model’s explanations.

### 5.1. Clinical Interpretation of Explainability Results

The interpretability analysis using SHAP [16] and LIME [17] reveals that the FL-LSTM framework bases its decisions on physiologically meaningful ECG features that align with established cardiological knowledge. For arrhythmic subjects, the model assigns the highest importance to the P-wave height (P-H), R-wave height (R-H), RR interval, and QRS duration. These features capture abnormalities in atrial and ventricular depolarization patterns, irregular beat spacing, and conduction block phenomena—hallmark characteristics of cardiac arrhythmia [9,25].

For ischemia cases, the most influential variables are the QT interval, corrected QT (QTc), ST-segment level, and R-wave amplitude. Prolonged QTc and depressed ST segments are classical electrophysiological indicators of myocardial ischemia or hypoxia, confirming that the model attends to clinically relevant morphological cues rather than spurious correlations [20]. In healthy recordings, SHAP and LIME highlight the stability of RR intervals, normal QRS widths, and balanced P–R wave amplitudes, all consistent with regular sinus rhythm and intact conduction pathways [41].

Overall, the explainability results demonstrate that the FL-LSTM framework preserves medical validity even under federated training. The global SHAP distributions correspond to known physiological hierarchies of importance, while the local LIME explanations provide case-by-case insights that clinicians can verify directly against ECG morphology. This synergy enhances the clinical trust and transparency of the proposed approach, bridging algorithmic interpretability with expert reasoning.

### 5.2. Computational Complexity and Real-Time Deployment

The proposed FL-LSTM framework was designed to remain lightweight and computationally feasible for integration into real-time clinical workflows. Each client model consists of three stacked LSTM layers (64, 32, and 16 units) followed by a fully connected softmax classifier, yielding fewer than one million trainable parameters. This compact design results in an average inference time of under 10 ms per ECG beat sequence on a single mid-range GPU (NVIDIA T4) and below 100 ms on a typical CPU workstation.

During federated training, computational effort is primarily consumed by the local update phase at each client. Because model aggregation via the Federated Averaging (FedAvg) algorithm involves only parameter summation and scaling, its communication cost grows linearly with model size and number of clients [52]. Hence, periodic model synchronization (every few minutes) is practical even in distributed healthcare environments.

Importantly, the proposed system operates on pre-extracted fiducial features rather than raw ECG waveforms. Most commercial ECG management systems (GE Muse™, Philips IntelliVue™, Biopac AcqKnowledge™) already compute these features as part of their standard diagnostic toolset. Therefore, the FL-LSTM module can be integrated as a plug-in for automated decision support without altering existing data pipelines or patient-data storage policies.

From a deployment standpoint, real-time inference can be executed locally within cardiology workstations, bedside monitors, or edge devices. Federated updates—typically requiring minutes rather than milliseconds—can be scheduled during off-peak hours or asynchronously to minimize clinical workflow disruption. In portable or wearable ECG systems, feature extraction and local inference could be performed on-device, while federated updates would occur periodically via secure cloud synchronization.

Although system-level issues such as client drop-outs, network latency, and secure aggregation are not explicitly modeled in this study [53], the small model footprint and low communication overhead suggest that real-world scalability is achievable. Future research will focus on expanding the model to larger, multi-institutional datasets and exploring adaptive communication strategies to further optimize training efficiency. This work contributes to advancing federated and explainable intelligence in cardiovascular diagnostics, paving the way for real-world deployment in distributed healthcare systems.

### 5.3. Limitations and Future Work

The proposed FL-LSTM framework shows strong performance and interpretability, yet several limitations remain. The study uses three public ECG datasets—MIT-BIH Arrhythmia, European ST-T, and FANTASIA—which, although widely used, do not reflect the full diversity of clinical practice. Variations in hardware, patient demographics, comorbidities, and real-time noise conditions are only partially represented. In addition, relying solely on fiducial features limits the ability to capture more subtle waveform patterns, and the federated setup assumes stable communication without accounting for latency, device heterogeneity, or client dropout.

Future work should incorporate more diverse multi-center datasets and clinical environments, including wearable ECG devices and bedside monitors [54]. Exploring hybrid architectures that combine interpretable fiducial features with raw-signal learning may allow the model to capture richer diagnostic information while maintaining transparency [55]. Techniques such as secure aggregation [53] and asynchronous federated updates could also improve robustness in real deployments.

Further research should include prospective clinical evaluations in real-time settings and extend the diagnostic scope to additional cardiac abnormalities. Collaborating closely with cardiologists will be essential to refine the explainability pipeline and ensure that model outputs remain clinically meaningful and actionable.

## 6. Conclusions

In this study, we proposed a Federated Learning-based Long Short-Term Memory (FL-LSTM) framework for multi-class classification of cardiac conditions using ECG fiducial features. The model successfully distinguishes among arrhythmia, ischemia, and healthy heart states while preserving patient privacy across decentralized datasets. By leveraging temporal sequence learning and federated aggregation through the FedAvg algorithm, the FL-LSTM model achieved high accuracy (92%), AUC (0.99), and F1 score (0.91) across three heterogeneous datasets, demonstrating robust generalization. The integration of explainable artificial intelligence techniques, namely SHAP and LIME, provided transparent insights into the influence of key ECG features such as P-wave height, R-wave height, QTc interval, and RR interval on model predictions. These interpretations not only align with established clinical knowledge but also enhance the trustworthiness and applicability of the model in real-world healthcare settings. Future work will extend the framework to larger multi-institutional datasets, incorporate additional ECG biomarkers, and optimize federated aggregation and communication protocols to improve efficiency. 

## Figures and Tables

**Table 1:** Population demographics of ECG datasets used in this study.

Dataset	Total Subjects	Male Subjects	Female Subjects	Age Range (Male)	Age Range (Female)
MIT-BIH Arrhythmia	47	25	22	32–89	23–89
European ST-T	79	71	8	30–84	55–71
FANTASIA	40	20	20	21–34 (young), 68–85 (elderly)	21–34 (young), 68–85 (elderly)

**Table 2:** Experimental Setup and Hyperparameters for Heart Disease Classification.

Parameter	Value/Description
Environment	Google Colab, PyTorch framework
GPU	NVIDIA Tesla T4, 15 GB
CPU	Intel Xeon, 2 cores, 2.30 GHz
RAM	13 GB
Dataset Split	67% training, 33% testing
Clients per round	10 clients (each with max 10% of training data)
Data Augmentation	SMOTE applied to training set only
Batch Size	32
Epochs	15
Learning Rate	0.001
Optimizer	Adam
Loss Function	Sparse Categorical Cross-Entropy

**Table 3:** Mean ± standard deviation of performance metrics over 10-fold federated cross-validation and final test set evaluation.

Model	Accuracy (%)	Precision	Recall	F1	AUC	*p*-Value
FL-LSTM	92.3 ± 0.7	0.91 ± 0.02	0.90 ± 0.03	0.91 ± 0.02	0.99 ± 0.01	–
Centralized LSTM	90.1 ± 0.8	0.88 ± 0.03	0.87 ± 0.04	0.88 ± 0.03	0.97 ± 0.02	<0.05

**Table 4:** Performance metrics of the FL-LSTM model over 15 training rounds.

Round	Accuracy	F1 Score	FNR	FPR	Loss	MC Rate	Precision	Recall
0	0.3336	0.1668	0.0000	1.0000	1.1331	0.6664	0.1113	0.3336
1	0.7133	0.7014	0.1891	0.1100	0.6420	0.2867	0.7792	0.7133
2	0.8444	0.8436	0.0596	0.1157	0.3861	0.1556	0.8566	0.8444
3	0.8650	0.8644	0.0342	0.1299	0.3443	0.1350	0.8700	0.8650
4	0.8738	0.8736	0.0506	0.1024	0.3135	0.1262	0.8784	0.8738
5	0.8939	0.8937	0.0372	0.0932	0.2877	0.1061	0.8959	0.8939
6	0.8990	0.8988	0.0318	0.0939	0.2760	0.1010	0.9007	0.8990
7	0.8887	0.8889	0.0874	0.0580	0.3035	0.1113	0.8910	0.8887
8	0.9007	0.9006	0.0443	0.0823	0.2731	0.0993	0.9024	0.9007
9	0.8974	0.8976	0.0649	0.0763	0.2790	0.1026	0.8991	0.8974
10	0.9015	0.9015	0.0531	0.0707	0.2665	0.0985	0.9034	0.9015
11	0.9033	0.9032	0.0511	0.0675	0.2625	0.0967	0.9036	0.9033
12	0.9039	0.9039	0.0436	0.0769	0.2852	0.0961	0.9052	0.9039
13	0.9011	0.9010	0.0391	0.0878	0.2674	0.0989	0.9027	0.9011
14	0.9023	0.9023	0.0559	0.0615	0.2627	0.0977	0.9040	0.9023
15	0.9091	0.9088	0.0347	0.0757	0.2446	0.0909	0.9101	0.9091

**Table 5:** Comparison of existing federated learning approaches for ECG-based heart disease classification. The proposed model uniquely performs multi-class classification (arrhythmia, ischemia, healthy), while others target only binary or single-disease tasks.

Study (Year)	FL Model/Architecture	Dataset/ECG Type	Performance	Classification Scope	XAI
Santos et al. (2024) [12]	FED CNN Architecture	PhysioNet Challenge [39]	F1 score 77%	Atrial Fibrillation	No
Yaqoob et al. (2022) [10]	Modified Artificial Bee Colony	Heart Disease Data Set (Cleveland) [40]	Accuracy 81%	Single: cardiac prediction	No
Khan et al. (2024) [13]	Asynchronous DNN	UCI and Switzerland	Accuracy 88%	Single: cardiac prediction	No
Alasmari et al. (2025) [11]	Asynchronous DNN	Cardiac images, ECG signals, patient records, and nutrition data	Accuracy 97%	Single: cardiac prediction	No
Zeleke et al. (2024) [9]	TCN + Attention	MIT-BIH ECG	Accuracy 91%	Single: Arrhythmia	Heatmap
Agrawal et al. (2024) [41]	DP-FedAvg (Differential Privacy-based FL)	Alberta Health Services (AHS)	Accuracy 81%	Binary: Healthy vs. Diseased	No
**Proposed Work (2025)**	**Federated LSTM**	**MIT-BIH, European ST-T, Fantasia**	**Acc. 92%, AUC 99%, F1 91%**	**Multi-class: Arrhythmia, Ischemia, Healthy**	SHAP and LIME

## Data Availability

Data will be made available on request.
